# Supplementary figures and images for: Parallel phospholipid transfer by Vps13 and Atg2 determines autophagosome biogenesis dynamics
Source: J Cell Biol. 2023 Apr 28;222(7):e202211039. doi: 10.1083/jcb.202211039 (PMC10148235; doi:10.1083/jcb.202211039)

**B**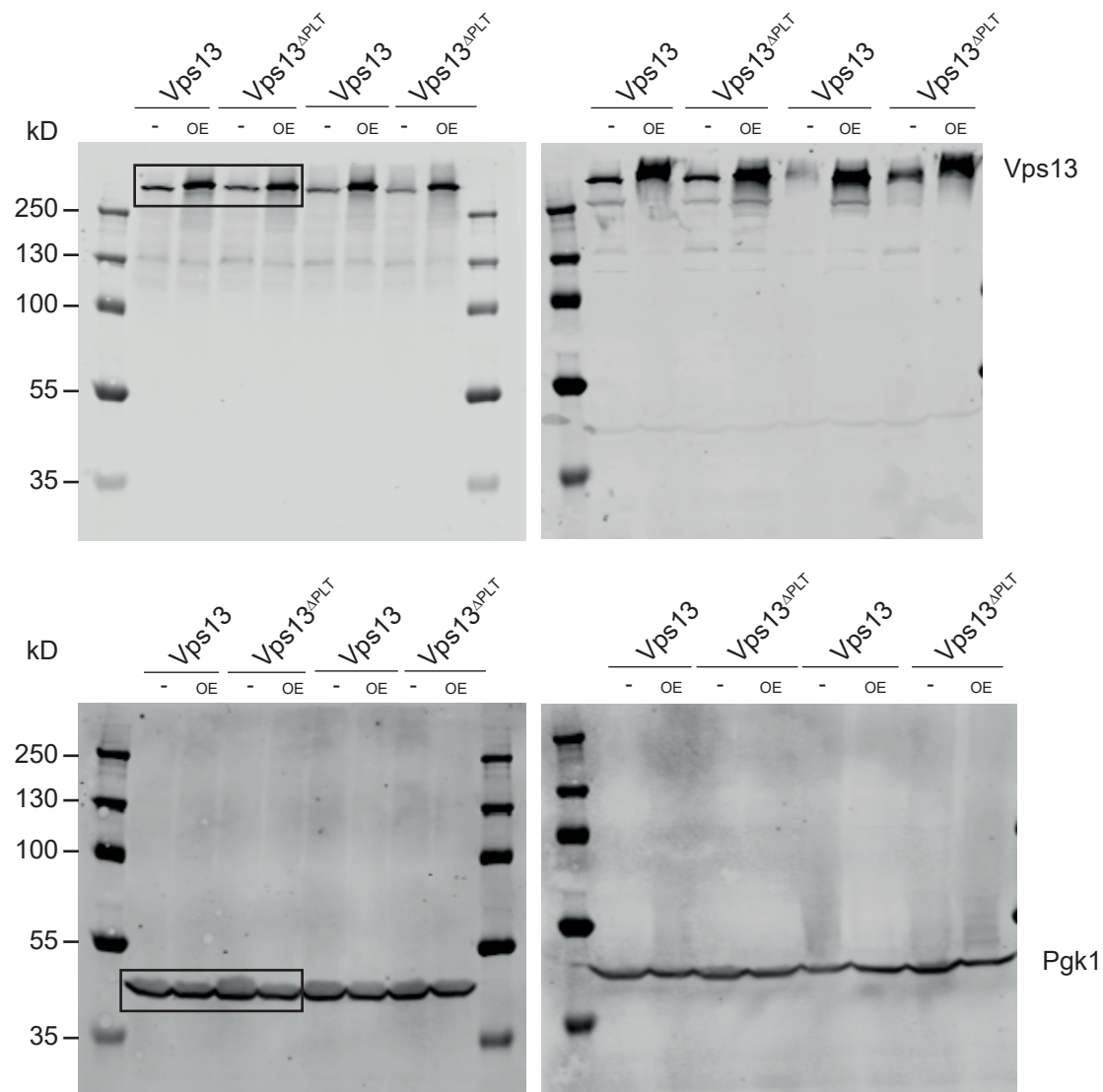

Supplement: SourceData F5 — is the source file for Fig. 5. [file JCB_202211039_SourceDataF5.pdf]

**D**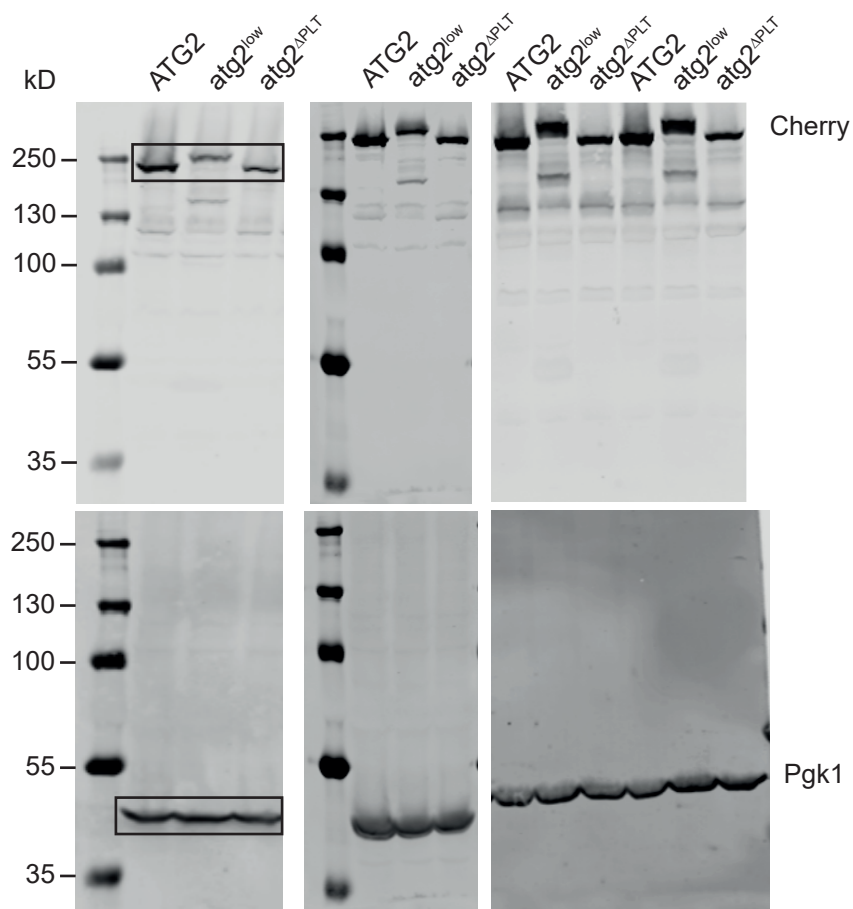**E**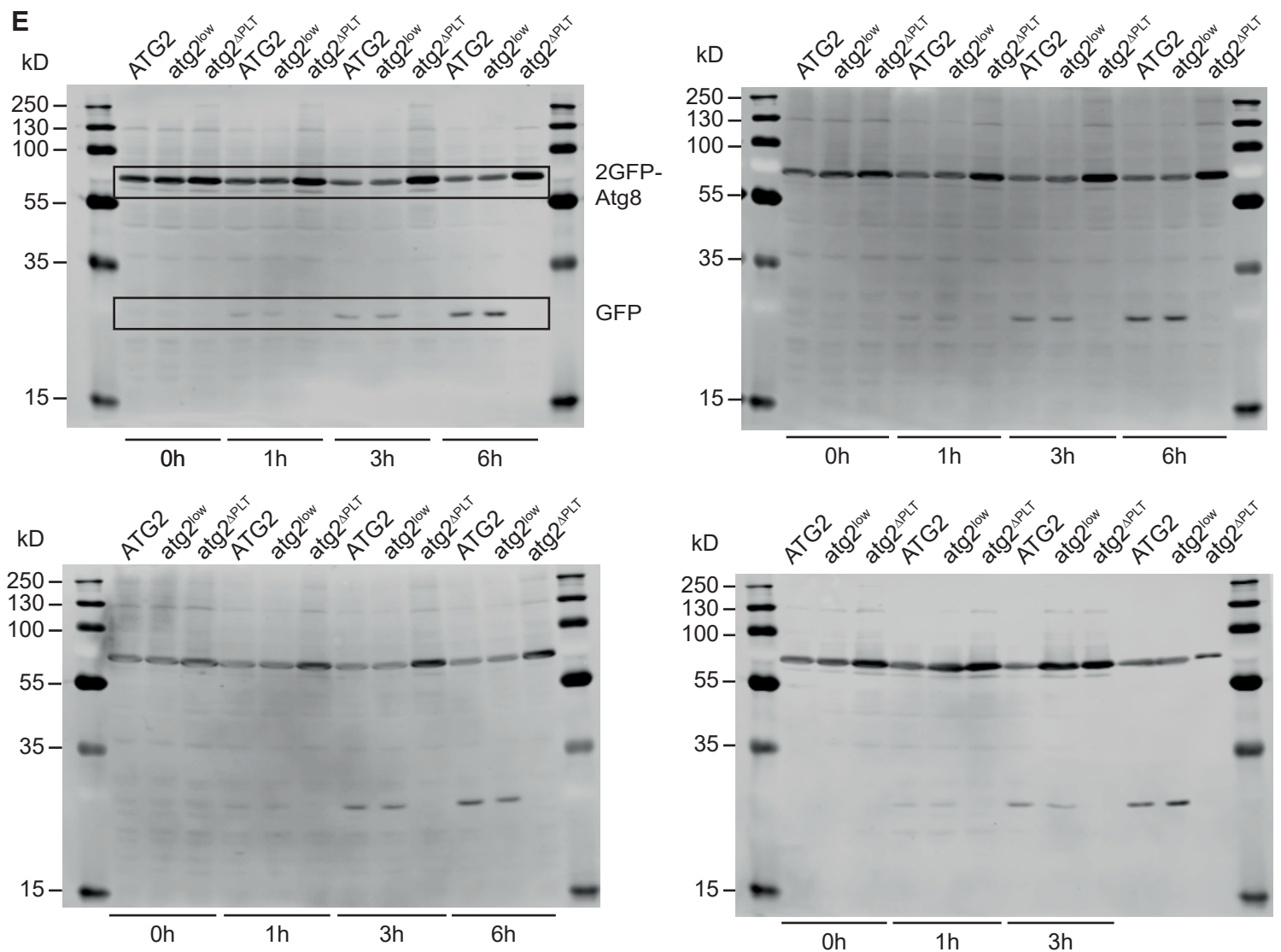

Supplement: SourceData FS1 — is the source file for Fig. S1. [file JCB_202211039_SourceDataFS1.pdf]

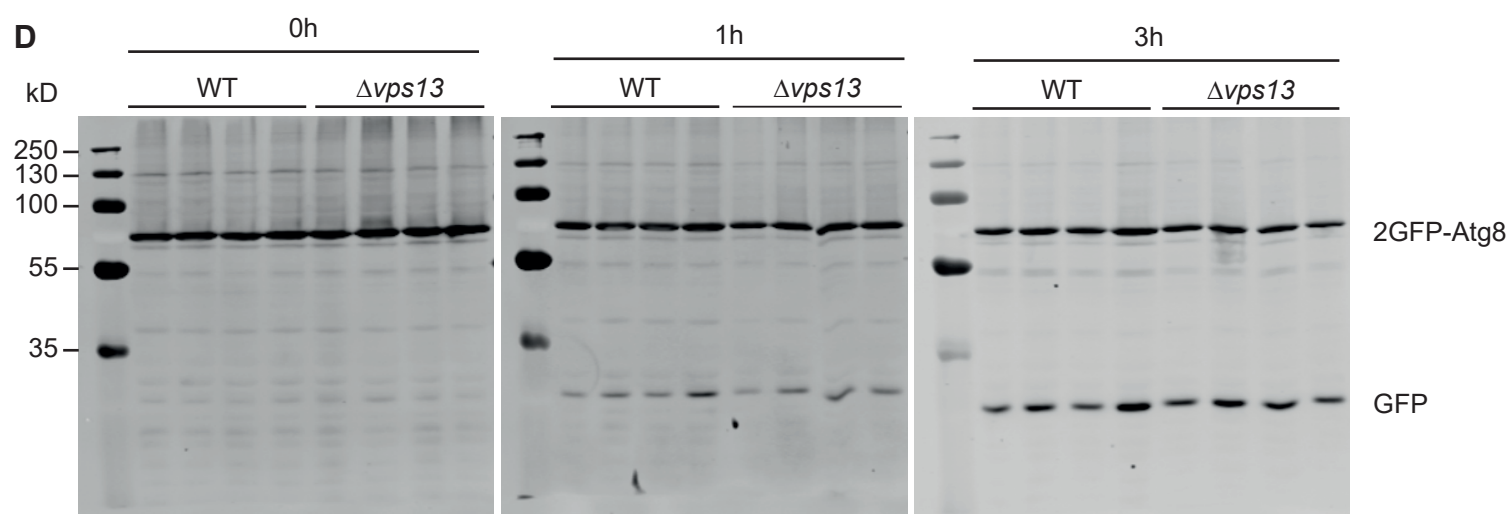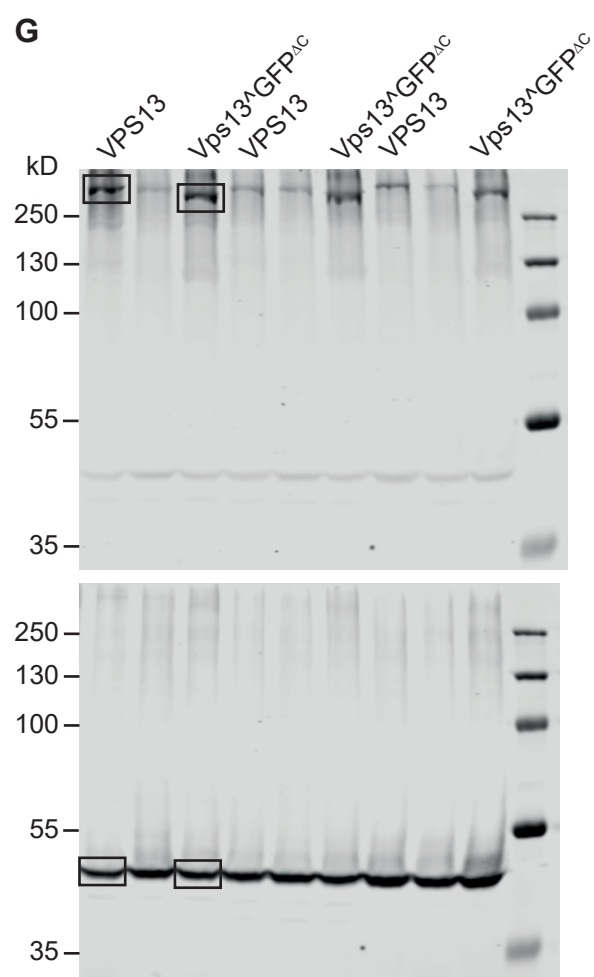

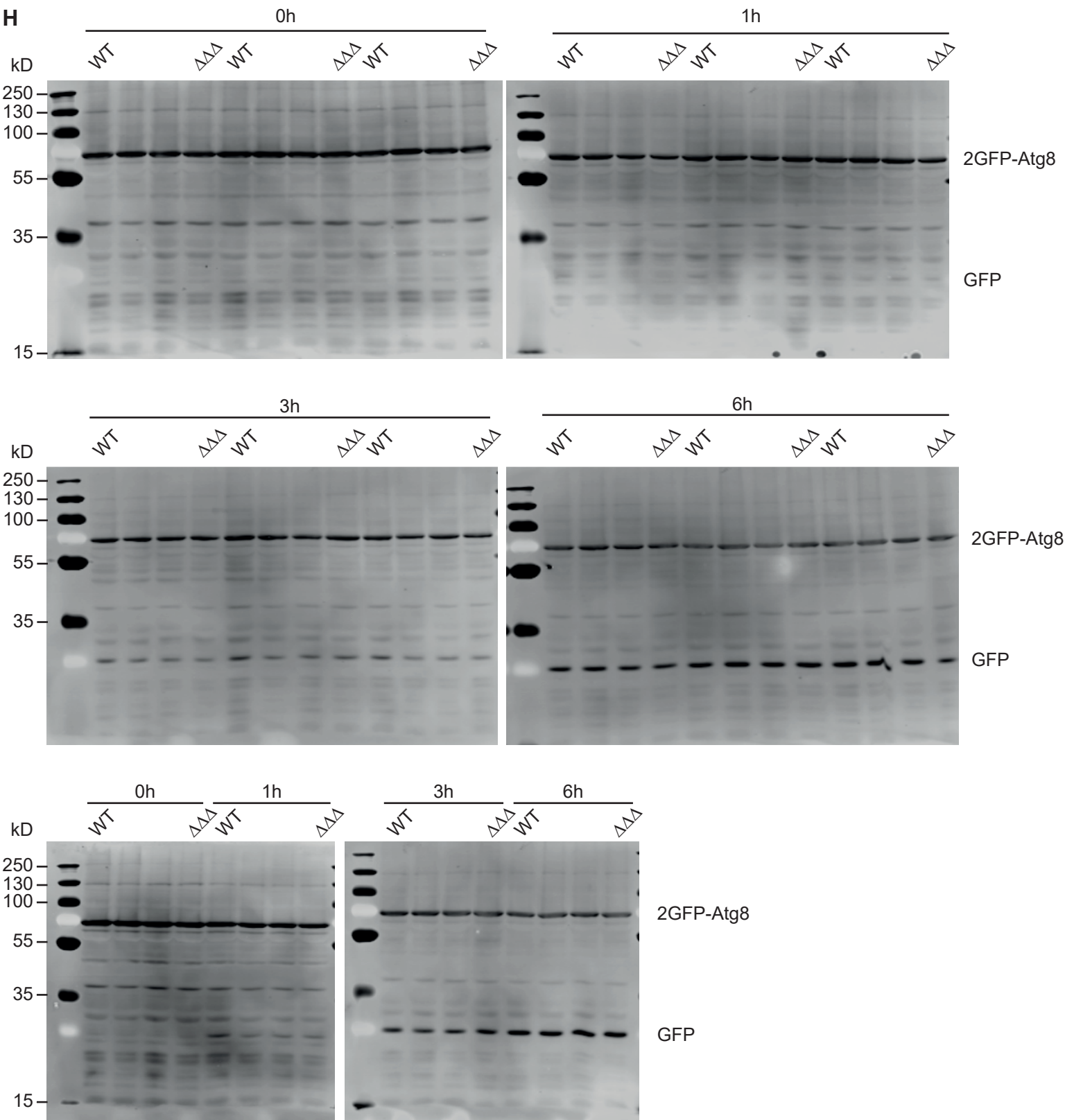

Supplement: SourceData FS2 — is the source file for Fig. S2. [file JCB_202211039_SourceDataFS2.pdf]

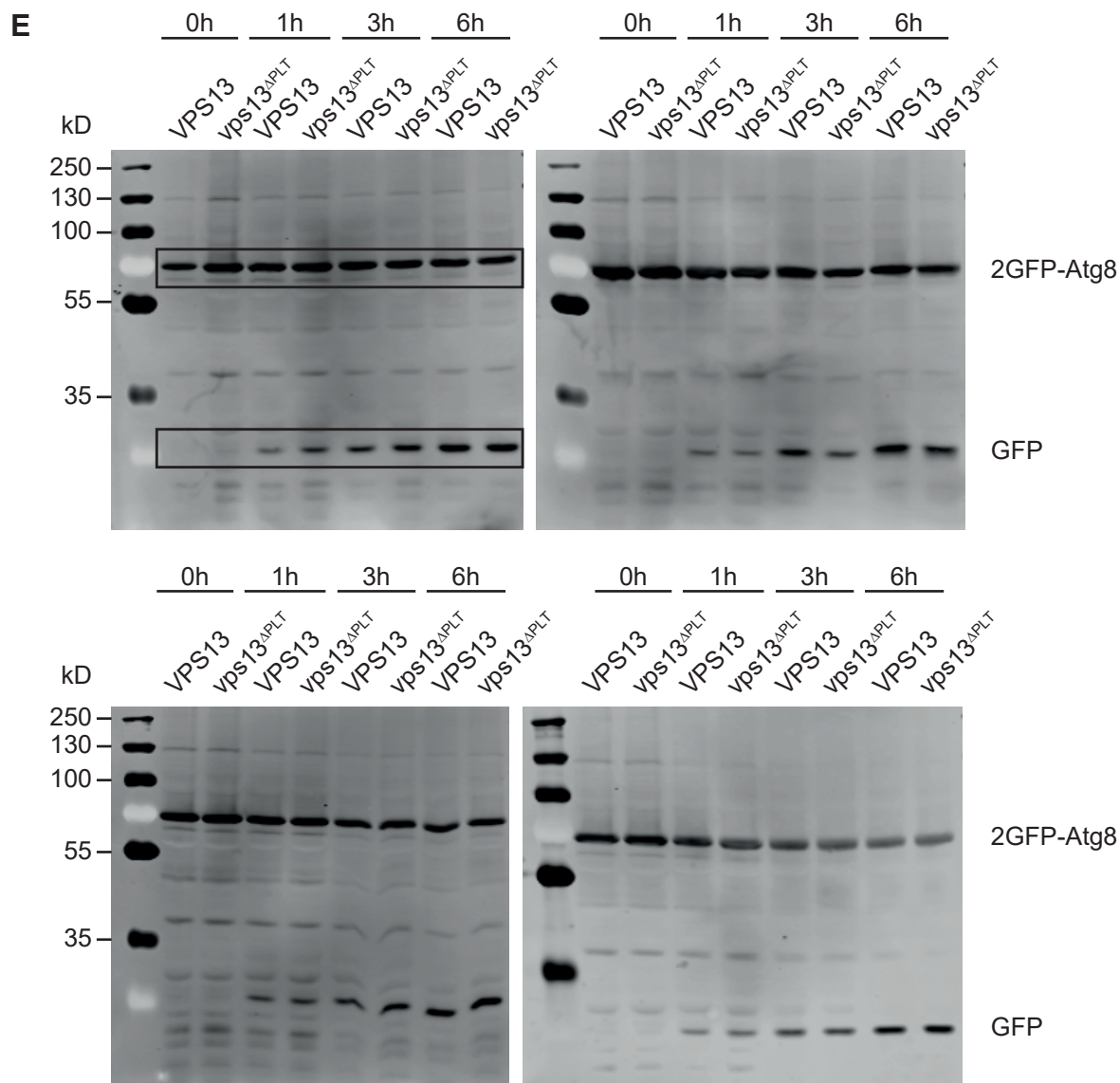

Supplement: SourceData FS3 — is the source file for Fig. S3. [file JCB_202211039_SourceDataFS3.pdf]
